# Does intraoperative contamination during primary knee arthroplasty affect patient-reported outcomes for patients who are uninfected 1 year after surgery? A prospective cohort study of 714 patients

**DOI:** 10.1080/17453674.2020.1811552

**Published:** 2020-09-01

**Authors:** Tobias Justesen, Jakob B Olsen, Anne B Hesselvig, Anne Mørup-Petersen, Anders Odgaard

**Affiliations:** Department of Orthopedic Surgery, Copenhagen University Hospital Herlev-Gentofte, Copenhagen, Denmark

## Abstract

Background and purpose — It is well recognized that some knee arthroplasty (KA) patients present with prolonged postoperative inflammation and some develop persistent pain. It can reasonably be speculated that some of these problems develop because of low-grade infections with low virulence bacteria caused by intraoperative contamination. This prospective study was performed to investigate whether intraoperative contamination results in lower patient-reported outcomes (PRO) for patients who were clinically uninfected in the first year after surgery.

Patients and methods — We combined data from 2 major prospective studies on patients undergoing primary KA at 2 Danish hospitals between September 2016 and January 2018. Pre- and postoperative (1.5, 3, 6, and 12 months) PROs and intraoperative microbiological cultures were obtained on a total of 714 patients who were included in the study. Based on the microbiological cultures, the patients were divided into 2 groups, contaminated and non-contaminated, and differences in PROs between the 2 groups were analyzed.

Results — 84 of 714 (12%) patients were intraoperatively contaminated; none of the 714 patients developed clinical infection. The preoperative Oxford Knee Score was 24 and 23 for contaminated and non-contaminated patients, respectively, improving to 40 and 39 at 1 year (p = 0.8). 1-year AUC for Oxford Knee Score and absolute improvement at each postoperative time point for Forgotten Joint Score and EQ-5D-5L also were similar between contaminated and non-contaminated patients.

Interpretation — Patient-reported outcomes from 714 patients do not indicate that intraoperative contamination affects the knee-specific or general health-related quality of life in primary KA patients who are clinically uninfected 1 year after surgery.

Intraoperative bacterial contamination occurs in up to one-fourth of joint replacement procedures (Byrne et al. 2007, Font-Vizcarra et al. 2011, Frank et al. 2011, Lindeque et al. 2014, Hesselvig et al. 2020). Periprosthetic joint infections (PJIs) causing clinical signs occur in approximately 1% of patients and it is suspected that most contaminations are of no clinical importance. Some microorganisms remain viable and dormant in biofilms with a potential of eliciting an inflammatory response leading to tissue destruction and pain (Zimmerli et al. 2004, Arnold et al. 2013, Antony and Farran 2016). It has been shown that bacteria are indeed present on a high percentage of implants (Jakobsen et al. 2018) and it has been speculated that they can be responsible for cases of aseptic loosening (Ribera et al. 2014, Rothenberg et al. 2017).

The immediate postoperative inflammatory response associated with recovering from the surgical injury (Bilgen et al. 2001) may be potentiated by the inflammatory response caused by bacterial contamination. It is well recognized that some patients present with prolonged postoperative inflammation, some develop persistent pain, and others develop a swollen and stiff joint and it can reasonably be speculated that some of these problems develop because of low-grade infections due to bacterial contamination.

Our hypothesis is that intraoperative contamination that does not result in an acute or delayed infection will result in a prolonged inflammatory response that causes increased discomfort and prolonged rehabilitation, which will be reflected in patient-reported outcomes (PROs).

To our knowledge only 1 study (Ibrahim et al. 2011) has looked into the relationship between intraoperative contamination and PROs. However, that study investigated patients undergoing hip arthroplasty and did not have any data for the first 8 postoperative years. We investigated whether intraoperative contamination in knee arthroplasties without subsequent clinical infection results in lower Oxford Knee Score (OKS), lower Forgotten Joint Score (FJS), or lower EQ-5D-5L score in the first year after surgery.

## ^Patients and methods^

The study design was according to STROBE guidelines. This prospective cohort study combines data from 2 prospective multicenter studies, ICON (Hesselvig et al. 2020) and SPARK (Mørup-Petersen et al., personal communication). These 2 studies enrolled patients from multiple hospitals over a 2-year period (2016–2018) with a partially shared patient cohort. The patients included in this study were enrolled in both the ICON and SPARK studies at 2 high-volume knee arthroplasty centers (Copenhagen University Hospital Gentofte and Aarhus University Hospital) between September 1, 2016 and January 1, 2018.

The ICON study included 1,187 patients who underwent primary knee arthroplasties. Patients were instructed to shower preoperatively on the day of the procedure using a normal body wash and no moisturizer afterwards. Local guidelines did not include any pre-admission skin or nasal decontamination. Before surgery, all patients were disinfected twice using a 0.5% chlorhexidine gluconate solution with 80% alcohol. Afterwards, around half of the patients (603 of 1,187) were draped with an antimicrobial incision drape (Ioban2, 3M Health Care, St. Paul, MN, USA). All surgeons routinely use 2 pairs of gloves (inner and outer gloves). According to local guidelines the surgeons changed the outer gloves after preparation of the surgical field, prior to handling the prosthesis, and when using bone cement. All cases of cemented knee arthroplasty were done with cement containing antibiotics (i.e., gentamycin); no vancomycin was placed within the knee/wound. All operations were performed in laminar airflow operation rooms and all patients were given prophylactic antibiotics consisting of either dicloxacillin 2 g or cefuroxime 1.5 g, depending on the hospital routine and allergy status of the patient. This information was not recorded by individual patient. Information on age, operation date, sex, location (left or right), and duration of surgery was collected.

Exposure data, i.e., intraoperative contamination, was obtained by 2 dry wound swabs (Copan Diagnostics Inc. Murrieta, CA, USA) from each patient and a wash from the surgeon’s glove during surgery. Both swabs were taken of the lateral wound edge, the first just after incision and the second swab just prior to closure of the skin. Approximately 30 minutes into the operation, prior to handling the prosthesis and possibly bone cement, the surgeon changed the outer gloves. The glove from the dominant hand was turned inside out and washed with 10 mL of isotonic saline. The samples were cultured according to Danish guidelines and susceptibility tested using Eucast breakpoints (eucast.org 2019). Identification was done using matrix-assisted laser desorption/ionization time-of-flight mass spectrometry (Maldi-Tof, Bruker Daltonics, Hamburg, Germany). Contamination was defined as any amount of bacterial growth from 1 or more of the swabs from either the wound edge or the surgeon’s glove, no matter the type of bacteria.

The SPARK study was an observational cohort study of 1,452 patients who underwent primary knee arthroplasty surgery. The patients completed a set of PRO questionnaires preoperatively and at 1.5, 3, 6, and 12 months postoperatively, sent by either email or letter (Procordo Software, Copenhagen, Denmark). The PRO set included OKS, FJS, and EQ-5D-5L. The inclusion and exclusion criteria are described in Table 1 (see Supplementary data).

The following outcomes were investigated: 1-year AUC for OKS changes from baseline, absolute differences in OKS and EQ-5D-5L between baseline and 1.5, 3, 6, and 12 months after surgery, and differences in absolute postoperative scores for FJS at 3, 6, and 12 months. The modified version of OKS was applied (0–48, 48 best). The FJS comprises 12 items (total score 0–100) with higher scores reflecting better outcomes. The EQ-5D-5L consists of the EQ visual analogue scale (VAS) and the EQ-5D-5L descriptive system. The EQ VAS records the patients’ self-rated health on a scale ranging from 0 to 100, 100 being the best. The EQ-5D-5L comprises 5 dimensions: mobility, self-care, usual activities, pain/discomfort, and anxiety/depression. The results from the 5 dimensions were converted into index values (–0.22 to 1, 0 corresponding to death, negative numbers health states worse than death, and 1 being perfect health) based on data from the Danish population (Janssen et al. 2013). To determine whether any absolute difference in changes from baseline in OKS between the contaminated and non-contaminated patients was likely to be perceived as relevant by the patients, the minimally important difference (MID) was used. The MID between the responses “a little better” and “about the same” was found to be 5 OKS points in a study by Beard et al. (2015). Our study is considered blinded because the patients were not informed whether they were contaminated or not.

### ^Statistics^

**Figure 1.**
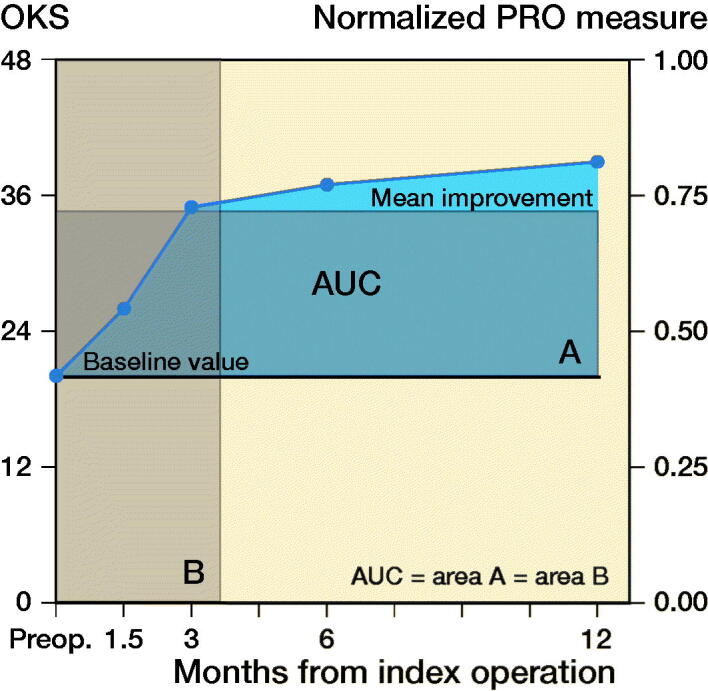
Example of area under the curve for a random patient in the study. The AUC is the blue area above the baseline. Full circles indicate values of OKS from 20 at baseline (preoperative value) to 39 at 12 months postoperatively. The AUC is the same size as rectangle A or B. A represents the average improvement in OKS during the first postoperative year by the left y-axis. B represents a translation of A into months with optimal (value 48) OKS.

Patients with missing postoperative data at 12 months or missing data at more than 2 time points were excluded from the analysis of AUC. AUC was calculated for PRO changes from baseline using the trapezium rule (Matthews et al. 1990) as an overall time-adjusted measure for changes in OKS. The x-axis (Figure 1) indicates time in months and the y-axis is a normalized PRO measure (0–1, no dimension), giving AUC the dimension “time (months)” (Odgaard et al. 2018). Since an MID for AUC has not been suggested, AUC data is presented as an equivalent measure of “gained months with optimal OKS” (value 48) for each group. When calculating the AUC for OKS we used linearly interpolated values for missing data at the time points 1.5, 3, and 6 months. We analyzed a subgroup of contaminated patients with 2 positive cultures. Analyses were done using a univariable model and multiple linear regression models when adjusting for confounders. The analyses included adjustment of the parameter estimates for differences in the distributions of sex, age, type of prosthesis, and duration of surgery. These variables are known to influence either contamination (Byrne et al. 2007, Hesselvig et al. 2020) or PRO improvements (Weber et al. 2018, Tolk et al. 2019), and based on clinical experience they may reasonably be suspected of being confounders. None of the variables can induce bias when adjusting for these, as neither exposure nor outcome can affect the variables (i.e., they cannot be either a mediator or collider) (Shrier and Platt 2008). P-values < 0.05 were considered statistically significant. Confidence intervals (CI) are defined as 95%. The analyses were performed using the SAS Enterprise Guide (version 7.15 HF3, SAS Institute, Cary, NC, USA).

### ^Ethics, registration, funding, and potential conflicts of interest^

Ethical approval was provided by the Regional Committee of Health Research Ethics (September 2, 2016, Jr. No. H-15012754) and data management was approved by the Danish Data Protection Agency (August 1, 2016, Jr. No. HGH-2016-087, I-Suite no: 04819). Permission to use the EQ-5D-5L questionnaires was given by the EuroQol Research Foundation (January 17, 2019, ID number 28583). All included participants gave informed consent.

The SPARK study was funded by the Health Research Fund of the Capital Region of Denmark and the ICON study was funded by 3M Health Care and the University of Copenhagen. However, this particular study did not receive any specific grant from funding agencies in the public, commercial, or not-for-profit sectors. Furthermore, no sponsors were involved in conduct of the research or preparation of the article.

AO is paid speaker by Stryker and DePuy, paid consultant by Stryker and DePuy, receives research support by Zimmer-Biomet, Stryker, and DePuy, and is Chairman for Danish Knee Arthroplasty Register.

## ^Results^

**Figure 2.**
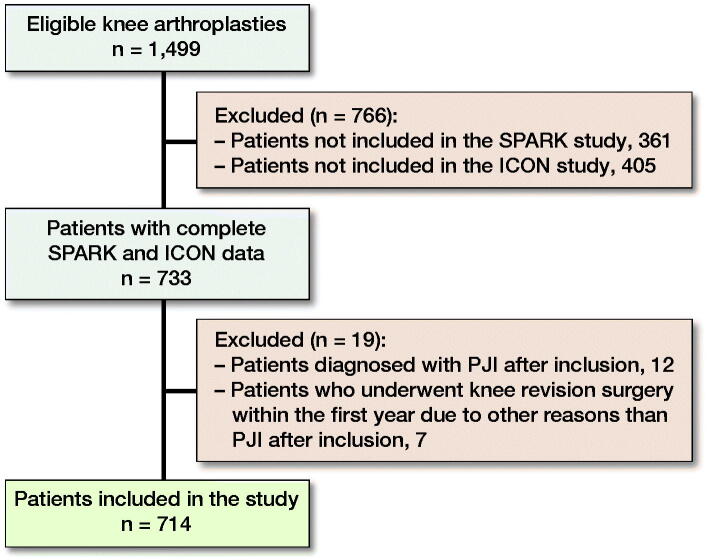
Flow diagram of the inclusion process. PJI = periprosthetic joint infection

At the start of analyzing data for this study May 2019, 1,499 patients were included in either the SPARK study, the ICON study, or both studies (Figure 2). 766 patients were only included in either the ICON or SPARK study due to different enrollment centers and enrollment periods and were thus excluded. 19 patients were excluded due to PJIs or revision surgery. 2 of the 12 patients excluded due to PJIs were intraoperatively contaminated. 1 patient was contaminated with *Micrococcus* species while joint fluid and biopsy at revision surgery showed *Streptococcus dysgalactiae.* The other patient was contaminated with *Staphylococcus capitis* and *epidermidis*, and joint fluid and biopsy at revision surgery revealed *Staphylococcus epidermidis.* None of the 7 patients who underwent revision surgery, for reasons other than PJIs, were intraoperatively contaminated. Furthermore, none of the intraoperative biopsies from the revisions, which were done on 4 of the patients on the slightest suspicion of infection, revealed any positive culture. The reasons for revision surgery were: rupture of the posterior cruciate ligament, medial tibial plateau fracture, instability, loosening of the prosthesis, progression of arthrosis, and in 2 cases pain and instability. A sufficient PRO sequence and contamination data were available for 714 patients (389 women and 325 men), who were included in the final analysis.

The types of knee arthroplasties included total knee arthroplasty (n = 510), medial unicompartmental knee arthroplasty (n = 170), lateral unicompartmental knee arthroplasty (n = 6), and patellofemoral arthroplasty (n = 28). 12% (84) of the patients were intraoperatively contaminated, 1.1% (8) had 2 or more contaminated samples, and 1.3% (9) were contaminated by more than one organism. Coagulase-negative staphylococci were the most common contaminating organisms (Table 2).

**Table 2.** Contaminating organisms and contaminated samples

		Sample	
Organism	Lateral wound edge after incision	Surgeon’s glove	Lateral wound edge prior to closure
Coagulase-negative staphylococci	23	19	36
*Micrococcus*	3	1	6
*Streptococcus*	5	0	2
Gram-positive rods	4	2	3
Gram-negative rods	1	0	1
*Staphylococcus aureus*	0	0	0

The patients had a mean age of 68 years (SD 9, range 28–93) and mean BMI was 29 (SD 5, range 18–52). The mean duration of surgery was 66 minutes (SD 15, range 30–130). Baseline data for age, BMI, OKS, EQ-5D-5L index value, EQ VAS, and type of prosthesis were similar between the contaminated and non-contaminated groups. The groups differed slightly regarding sex and duration of surgery (Table 3), i.e., male sex and longer operation time were associated with increasing contamination.

**Table 3.** Univariable analysis of patients regarding negative vs. positive intraoperative cultures. Values are mean (standard deviation) unless other­wise specified

	Intraoperative cultures		
	negative	positive	
Variables	n = 630	n = 84	p-value
Age	68 (9)	67 (9)	0.8
Women, n (%)	352 (56)	37 (44)	0.04
BMI	29 (5)	29 (5)	0.9
Duration of surgery, min	65 (15)	69 (15)	0.01
Total knee arthroplasty, n (%)	445 (71)	65 (77)	0.2
Baseline OKS	23 (7)	24 (6)	0.5
Baseline EQ-5D-5L index value	0.6 (0.1)	0.6 (0.1)	0.9
Baseline EQ VAS	62 (22)	62 (20)	0.7

BMI = body mass index; OKS = Oxford Knee Score (0–48).

The patient-reported outcomes OKS, FJS, and EQ-5D-5L did not differ statistically significantly between the contaminated and non-contaminated groups when analyzed by AUC, absolute values at any of the postoperative time points, or by absolute differences from baseline (Tables 4, 5, and 6 [for Tables 5 and 6, see Supplementary data]).

**Table 4.** Univariable analysis: measures of change in Oxford Knee Score for contaminated vs. non-contaminated patients. Values are number of patients and absolute difference unless otherwise specified and (95% confidence interval)

	1.5 months	3 months	6 months	1 year	AUC 1 year ^a^
Non-contaminated	586 4.2 (3.6–4.8)	580 10.2 (9.6–10.9)	580 13.7 (13.1–14.4)	570 15.6 (15.0–16.3)	544 2.9 (2.7–3.0) ^b^
Contaminated	77 4.2 (2.7–5.7)	73 10.2 (8.3–12.0)	75 13.0 (11.0–15.1)	78 15.4 (13.6–17.1)	70 2.8 (2.4–3.2) ^b^
p-value	1.0	1.0	0.5	0.8	0.7

**^a^** 1-year area under the curve for the difference in OKS from baseline;

**^b^** number of gained months with optimal (48) OKS.

Mean 1-year OKS for the contaminated and non-contaminated groups was 40 (SD 6) and 39 (SD 8), respectively. The effect sizes of contamination on absolute differences in OKS between baseline and 1.5, 3, 6, and 12 months after surgery ranged from 0.01 to 0.70 (CI –2.0 to 2.7). All effect sizes were statistically insignificant and the range of the effect sizes was not greater than the minimally important difference (MID) of 5.

Furthermore, all outcomes were assessed by multiple linear regression models adjusting for sex, age, type of prosthesis, and duration of surgery. All p-values were still insignificant (range 0.4–1.0) and results were consistent with the unadjusted values.

Patients with 2 or more positive samples were analyzed using a univariable model and compared with the non-contaminated patients. The effect size of contamination regarding 1-year AUC (OKS) was 0.3 (CI –0.9 to 1.5), 0.5 to 0.9 (CI –5.3 to 7.1) regarding OKS changes between baseline and 1.5, 3, 6, and 12 months after surgery, and –1.1 to 7.3 (CI –19 to 26) regarding FJS at 3, 6, and 12 months. The same subgroup of patients was analyzed using a multiple linear regression model as well, which revealed results consistent with the univariable analyses. None of the analyses showed any statistically significant difference between the subgroup and the non-contaminated group. 

## ^Discussion^

Some primary knee arthroplasty patients experience prolonged postoperative inflammation, persistent pain, or a swollen and stiff joint. Our hypothesis was that some of these problems develop because of bacterial contamination, but which has not resulted in a clear-cut clinical infection. To our knowledge, this hypothesis has not been tested previously.

There is no standardized way of collecting data on intraoperative contamination during knee surgery. Similar studies have used 1 to 3 swab samples collected from a range of locations such as knife blades, suction tips, suture lines, the subcutaneous tissue when closing, fluid residues, and the splash basin (Byrne et al. 2007, Frank et al. 2011, Fuchs et al. 2018). The swab cultures used in this study are less sensitive in detecting intraoperative contamination than for example tissue samples (Aggarwal et al. 2013). This study does not account for any contamination that might occur postoperatively through the non-healed wound or by bacteremia (Zimmerli 2006). Only 2 of 12 patients excluded due to PJIs were intraoperatively contaminated, while the bacteria found during revision surgery in 1 of these cases matched the intraoperative contamination. These results are to be seen in relation to the above-mentioned limitations and the fact that no former studies have been able to prove a correlation between intraoperative contamination and subsequent infection (Davis et al. 1999, Byrne et al. 2007, Jonsson et al. 2014). None of the symptoms in 7 patients, who underwent revision surgery for reasons other than PJI, can be readily explained by bacteria, since none of them were intraoperatively contaminated and in the 4 cases where intraoperative biopsies from the revisions were done, none of the biopsies revealed any positive culture.

The contamination rate of 12% is within the range found in similar studies (Byrne et al. 2007, Font-Vizcarra et al. 2011, Frank et al. 2011, Lindeque et al. 2014) and in between the 10% (use of antimicrobial drape) and 15% (no use of antimicrobial drape) found in the study by Hesselvig et al. (2020) (for 363 of the 714 included patients antimicrobial drapes were used). Average age and sex distribution of included patients were comparable to those reported in the Danish Knee Arthroplasty Register (DKR), which has a completion rate of 95%.

The analyses of the patient-reported outcomes were based on the AUC analysis of OKS and complementing analyses of all PROs (OKS, FJS, EQ-5D-5L index value, and EQ VAS) at different sequential time points. All PRO results at baseline and postoperatively in our study were of the same magnitude as those found in similar knee arthroplasty studies (Nerhus et al. 2012, Hamilton et al. 2017, Bilbao et al. 2018, Odgaard et al. 2018). We found similar PRO scores in the contaminated and non-contaminated groups. Thus, the enhanced inflammatory response that intraoperative contamination hypothetically could cause was not severe or prolonged enough to significantly potentiate the general postoperative inflammatory response and to be reflected in the PROs. Our results do not support the hypothesis that intraoperative contamination, not resulting in an acute or delayed infection, would lead to increased discomfort in the first postoperative year.

The results in our study are in line with a former case-control study (Ibrahim et al. 2011), which did not find a correlation between contamination of the femoral head during hip replacement surgery and the patient-reported outcome measures Oxford Hip Score and EQ-5D. Since no other studies have investigated whether intraoperative contamination during knee or hip surgery is associated with lower PROs, our study brings new and needed data on the patient consequences of intraoperative contamination in arthroplasty surgery.

The cohort of 714 patients who underwent primary knee arthroplasty surgery will be further followed with the purpose of investigating the possible association of intraoperative contamination with late infections, aseptic loosening of prostheses, and revision surgery for reasons other than infections and loosening. So far, no such associations have been established within the first year after surgery.

In summary, patient-reported outcomes from 714 patients do not indicate that intraoperative contamination affects the knee-specific or general health-related quality of life in primary knee arthroplasty patients who are clinically uninfected within the first year after surgery.

## Supplementary Material

Supplemental Material

## Supplementary data

Tables 1, 5, and 6 as supplementary data in the online version of this article, http://dx.doi.org/ 10.1080/17453674.2020.1811552
