# Supplementary material for: Does intraoperative contamination during primary knee arthroplasty affect patient-reported outcomes for patients who are uninfected 1 year after surgery? A prospective cohort study of 714 patients
Source: Acta Orthop. 2020 Sep 1;91(6):750–5. doi: 10.1080/17453674.2020.1811552 (PMC8023922; doi:10.1080/17453674.2020.1811552)
Supplement: Supplemental Material [file IORT_A_1811552_SM3973.pdf]

Supplementary data

Table 1. Combined inclusion and exclusion criteria for the ICON and SPARK studies

Inclusion criteria
- Elective primary KA surgery of any type from September 1, 2016 to January 1, 2018
- > 18 years old

Exclusion criteria

Patient-specific conditions
- Hemophilia
- Dementia
- Language barriers with no possibility of translation by relatives
- Patients without an email address [a]
- Iodine allergy [b]
- Any antibiotic intake 4 weeks prior to surgery

Knee-specific conditions
- Previous open knee surgery
- Previous septic arthritis in the knee joint
- Knee prosthesis due to malignant tumor
- Severe developmental deformities of the knee

[a] This was only the case for the first two-thirds of the inclusion period. In the last third of the inclusion period, patients without an email address were included as well, and questionnaires were sent by letter.
[b] Patient reported.

Table 5. Univariable analysis: measures of change in Forgotten Joint Score for contaminated vs. non-contaminated patients. Values are number of patients and absolute score (95% confidence interval)

	3 months		6 months		12 months	
Non-contaminated	563	43.6 (41.4–45.7)	544	53.6 (51.3–55.8)	568	60.1 (57.9–62.4)
Contaminated	71	42.5 (36.6–48.3)	66	50.9 (44.8–57.1)	77	57.1 (51.4–62.8)
p-value		0.7		0.5		0.4

Table 6. Univariable analysis: measures of change in EQ-5D-5L and EQ-VAS for contaminated vs. non-contaminated patients. Values are number of patients and absolute improvement from baseline (95% confidence interval)

	1.5 months		3 months		6 months		12 months	
EQ-5D-5L index value								
Non-contaminated	514	0.1 (0.1–0.1)	566	0.2 (0.2–0.2)	559	0.2 (0.2–0.2)	579	0.2 (0.2–0.2)
Contaminated	61	0.1 (0.07–0.1)	72	0.2 (0.1–0.2)	68	0.2 (0.2–0.2)	77	0.2 (0.2–0.3)
p-value		0.7		0.5		0.9		1.0
EQ-VAS								
Non-contaminated	512	9.8 (8.0–11.7)	564	14.0 (12.2–15.8)	559	15.6 (13.8–17.3)	578	15.6 (13.8–17.4)
Contaminated	62	10.9 (5.7–16.1)	72	14.5 (9.8–19.2)	68	15.0 (10.0–20.0)	78	17.5 (12.5–22.5)
p-value		0.7		0.9		0.8		0.5
